# Clinical and genetic characteristics in pancreatic cancer from Chinese patients revealed by whole exome sequencing

**DOI:** 10.3389/fonc.2023.1167144

**Published:** 2023-05-29

**Authors:** Yonggang He, Wen Huang, Yichen Tang, Yuming Li, Xuehui Peng, Jing Li, Jing Wu, Nan You, Ling Li, Chuang Liu, Lu Zheng, Xiaobing Huang

**Affiliations:** ^1^ Department of Hepatobiliary, The Second Affiliated Hospital of Army Medical University, Chongqing, China; ^2^ Department of Medicine, Yinfeng Gene Technology Co Ltd, Jinan, China

**Keywords:** pancreatic ductal adenocarcinoma (PDAC), whole-exome sequencing, gene mutation, KRAS, TMB, PD-L1

## Abstract

**Background:**

Pancreatic ductal adenocarcinoma (PDAC) is one of the most fatal malignancies worldwide, mostly as a result of the absence of early detection and specific treatment solutions. Consequently, identifying mutational profiles and molecular biomarkers is essential for increasing the viability of precision therapy for pancreatic cancer.

**Methods:**

We collected blood and tumor tissue samples from 47 Chinese pancreatic cancer patients and used whole-exome sequencing (WES) to evaluate the genetic landscape.

**Results:**

Our results showed the most frequently somatic alteration genes were KRAS (74.5%), TP53(51.1%), SMAD4 (17%), ARID1A (12.8%), CDKN2A (12.8%), TENM4 (10.6%), TTN (8.5%), RNF43(8.5%), FLG (8.5%) and GAS6 (6.4%) in Chinese PDAC patients. We also found that three deleterious germline mutations (ATM c.4852C>T/p. R1618*, WRN c.1105C>T/p. R369*, PALB2 c.2760dupA/p. Q921Tfs*7) and two novel fusions (BRCA1-RPRML, MIR943 (intergenic)-FGFR3). When compared to the Cancer Genome Atlas (TCGA) database, there is a greater mutation frequency of TENM4 (10.6% vs. 1.6%, *p* = 0.01), GAS6(6.4% vs. 0.5%, *p* = 0.035), MMP17(6.4% vs. 0.5%, *p* = 0.035), ITM2B (6.4% vs. 0.5%, *p* = 0.035) and USP7 (6.4% vs. 0.5%, *p*= 0.035) as well as a reduced mutation frequency of SMAD4 (17.0% vs. 31.5%, *p* = 0.075) and CDKN2A (12.8% vs. 47.3%, *p* < 0.001) were observed in the Chinese cohort. Among the 41 individuals examined for programmed cell death ligand 1(PD-L1) expression, 15 (36.6%) had positive PD-L1 expression. The median tumor mutational burden (TMB) was found to be 12muts (range, 0124). The TMB index was higher in patients with mutant-type KRAS MUT/TP53 MUT (*p* < 0.001), CDKN2A (*p* = 0.547), or SMAD4 (*p* = 0.064) compared to patients with wild-type KRAS/TP53, CDKN2A, or SMAD4.

**Conclusions:**

We exhibited real-world genetic traits and new alterations in Chinese individuals with cancer of the pancreas, which might have interesting implications for future individualized therapy and medication development.

## 1 Introduction

Pancreatic ductal adenocarcinoma (PDAC) is one of the fatal malignant tumors, with a 5-year overall survival (OS) rate of smaller than 10percent (1). Carcinoma of the pancreas is the 6^th^ major cause of cancer mortality in China, and its global occurrence rate has risen dramatically in the recent decade (2). Even though nab-paclitaxel with FOLFIRINOX and the gemcitabine chemotherapy regimens have improved survival in an individual with pancreatic tumor (3, 4); Though, creating more effective medicines remains difficult. Hereditary variability is frequent in individuals with pancreatic tumor (5). Consequently, optimizing antitumor therapy selection depending on genetic variants is a major difficulty (6).

Earlier research has shown the genetic landscape of PDAC. For instance, previous studies have conducted comprehensive molecular characterizations in PDAC, including genomic modification of KRAS, CDKN2A, SMAD4, and TP53, driver genes (7, 8). However, most studies have been undertaken on participants from Western nations or have concentrated on the prognostic usefulness of gene change (9). The genetic landscape of Chinese PDAC remains unknown. One latest research investigated genomic characterization in Chinese participants with pancreatic cancer (10). However, the researchers utilized panel-based next-generation sequencing (NGS) to focus on the core DNA damage response (DDR) gene alterations. Other research has shown the genetic features of Chinese individuals with pancreatic tumor; therefore, reflecting the real-world genetic features of a pancreatic tumor in China is difficult due to an absence of blood matched germline or based on limited cases (11–13). To study the function of genomics, we studied 47 Chinese individuals with pancreatic cancer utilizing WES to investigate real-world genetic abnormalities and assess their possible clinical importance, which might play a growing role in PDAC precision medicine.

## 2 Methods

### 2.1 Patients and specimens

We collected blood and tumor tissue specimens from 47 individuals with surgically resected primary PDAC. The patients were treated at The Second Affiliated Hospital of Army Medical University between January 2019 and November 2022. Before specimen collection, all individuals gave signed informed permission. The Hospital’s ethics committee authorized this investigation. Whole-Exome Sequencing (WES) was used to conduct an NGS assessment of tumor DNA in formalin-fixed paraffin-embedded (FFPE) specimens in a Clinical Laboratory Improvement Amendments-certified and College of American Pathologists-accredited laboratory. The clinicopathologic characteristics (age, sex, stage, smoking and drinking) were collected.

### 2.2 Whole-exome sequencing and DNA extraction

For DNA extraction, tissue blocks were dissected using a macrodissection technique. DNA was extracted from formalin-fixed, paraffin-embedded (FFPE) samples utilizing QIAamp DNA FFPE Tissue Kits (Qiagen, Duesseldorf, Germany), and the quality of isolated genomic DNA was assessed utilizing Qubit ^®^ DNA Assay Kits (DNA concentration) and a Qubit 2.0 Fluorometer (Thermo Fisher Scientific, Carlsbad, CA, USA), and by 1% agarose gel electrophoresis. (assess DNA degradation). Hydrodynamic shearing (M220 Focused-ultrasonicator; Covaris, Woburn, MA, USA) generated 180-280 bp DNA fragments from 0.6g genomic DNA. Following the manufacturer’s instructions, sequencing libraries were constructed using an Agilent SureSelect Human All Exon V6 kit (Agilent Technologies, Santa Clara, CA, USA). Next, the index-coded library samples were clustered on an Illumina cBot Cluster Generation System, and the DNA libraries were sequenced on an Illumina HiSeq 2000 system.

### 2.3 Reads mapping and variation detection

The original fluorescence image files were used to generate raw data for base calling, and sequence artifacts and low-quality reads were removed to generate clean data. Afterwards, the data were aligned to the human reference genome (GRCh37/hg19) using Burrows Wheeler Aligner (BWA v0.7.15) with the default parameters (14). Then, SAMtools and Picard were utilized to classify BAM files and perform duplicate marking, local realignment, and quality recalibration at the base level (15). MuTect (16) was used to identify somatic single-nucleotide variants (SNVs), and IndelRealigner and RealignerTargetCreator in GATK (v1.0.6076) were used to call somatic insertions and deletions (indels) (17). According to the ClinVar database maintained by the National Center for Biotechnology Information, the deleterious variations associated with cancer.

### 2.4 Staining of PD-L1

The PD-L1 IHC 22C3 pharmDx assay performed immunohistochemistry (IHC) staining for PD-L1 expression. A tumor percentage score (TPS) of ≥1% was used to determine PD-L1 positivity.

### 2.5 Statistical analysis

SPSS software was used for statistical analysis. The Fisher’s exact test and one-way ANOVA were employed to investigate the relationships between clinical data and genetic features. A p-value of <0.05 was regarded as significant.

## 3 Results

### 3.1 Patient pathological and clinical features

**Figure 1:**
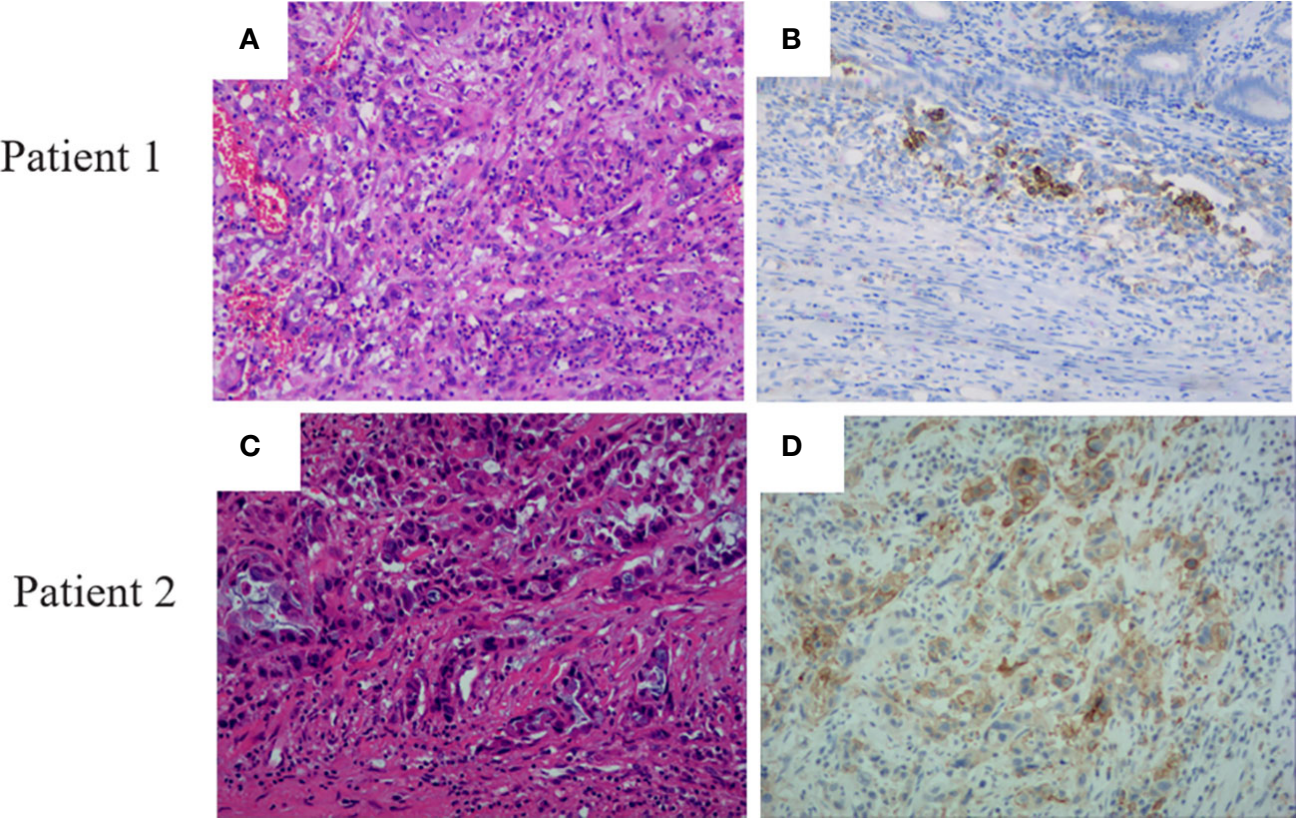
Pancreatic ductal adenocarcinoma patients’ H&E staining and PD-L1 expression. **(A)** HE staining of patient 1. **(B)** PD-L1 expression with a TPS expression of 50 in patient 1. **(C)** HE staining of patient 2. **(D)** PD-L1 expression with a TPS expression of 60 in patient 2.

A total of 47 PDAC individuals who have undergone the WES sequence participated in this investigation, containing 29 (62%) male and 18 female (38%) patients (**Table 1**). The median age was 58 (range, 29-75). According to accumulating research, TMB may be a viable biomarker for predicting the result of PD-1/PD-L1 suppressor in different malignancies (18). Cancer patients with high TMB might have more neoantigens than the host immune system can identify (18). The median TMB in this investigation was 12 mutants (range, 0-124) (**Table 1**). In addition, we investigated PD-L1 expression in PDAC patients. On a total of 41 PDAC samples, the expression of PD-L1 was evaluated. 15 (36.6%) of these individuals exhibited positive expression (TPS 1%), including two patients with extremely positive expression (TPS 50-60%) (**Figure 1**), whereas 26 (63.4%) exhibited negative expression (TPS 1%).

**Table 1:** Clinicopathologic features.

Characteristics		
Sample, n		47
Age, median (range)		58 (29-75)
Sex, n (%)		
Male		29 (62%)
Female		18 (38%)
PD-L1, n (%)		
TPS	NA	6 (13%)
	<1%	26 (55%)
	1-49%	13 (28%)
	≥50%	2 (4%)
MSI, n (%)		
MSI-H		0 (0%)
MSS		47 (100%)
TMB, median (range)		12 (0-124)

MSI-H, microsatellite instability-high; MSS, microsatellite stability; TMB-H, tumor mutational burden-high; TMB-L, tumor mutational burden-low; NA, non applicable.

### 3.2 Somatic and germline mutations in PDAC

**Figure 2:**
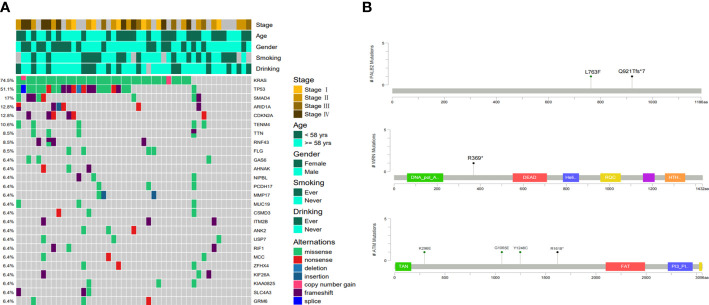
Molecular characteristics of Chinese Pancreatic ductal adenocarcinoma. **(A)** The somatic mutation landscape in Chinese pancreatic ductal adenocarcinoma patients. **(B)** Locations of pathogenic germline mutations in PALB2, WRN and ATM genes.

**Figure 3:**
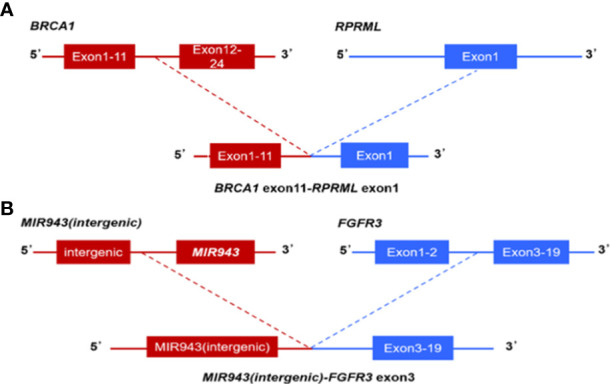
Next-generation sequencing findings BRCA1-RPRML and MIR943(intergenic)-FGFR3 fusion. **(A)** Schematic representation of BRCA1-RPRML fusion, this variant was generated by the fusion between exons 11 of BRCA1 and exons 3-19 of RPRML. **(B)** Schematic representation of the MIR943 intergenic region FGFR3 fusion, this variant was generated by the fusion of intergenic region between MIR943 and C4orf8 with exons 3-19 of FGFR3.

In 47 PDAC patients, the most prevalent somatic changes were observed in KRAS (74.5%), TP53 (51.1%), SMAD4 (17%), ARID1A (12.8%), CDKN2A (12.8%), TENM4 (10.6%), TTN (8.5%), RNF43 (8.5%), FLG (8.5%) and GAS6 (6.4%) (**Figure 2A**). Most KRAS mutations were missense mutations, and only two variations were copy number gain (CNV). Moreover, all of the TP53 mutations detected were projected to be harmful. For CDKN2A, all mutations were categorized as oncogenic. Furthermore, KRAS driver alterations and their somatic evolution correlations, SMAD4 and TP53 were investigated. Among all PDAC participants, 5 had variations in all 3 genes, and 35 had a mutation in 2 genes, KRAS and TP53. In addition, two novel BRCA1-RPRML and MIR943 (intergenic)-FGFR3 fusions were detected (**Figures 3A, B**). Exons generated one fusion 11 of BRCA1 and exons 3-19 of RPRML, and the other was generated by chr4:1801213 (the intergenic region between MIR943 and C4orf8) and exons 3-19 of FGFR3. Besides, we can differentiate between somatic and germline alterations using matching healthy tissue as a control. Among the 47 participants, 3 (6.4%) had a harmful germline mutation. **Table 2** details three participants with deleterious germline mutations. Moreover, the three mutated genes were all associated with DNA damage response. They play an important role in DNA repair, replication, transcription and telomere maintenance. The patient with an ATM c.4852C>T/p. R1618* mutation was a 61-year-old female. A 55-year-old female patient carries a germline WRN c.1105C>T/p. R369* mutation. Another harmful germline mutation in the PALB2 c.2760dupA/p. Q921Tfs*7 gene (**Figure 2B**), which contributes to 3-4percent of incidences of familial pancreatic tumor (19), was detected in a 29-year- old female patient. PALB2 germline mutation increases the risk of breast and pancreatic tumors.

**Table 2:** Deleterious germline mutations in pancreatic cancer patients.

Patient ID	Sex	Age	Gene	c. change	AA change
P17	Female	29	PALB2	c.2760dupA	p. Q921Tfs*7
P32	Female	55	WRN	c.1105C>T	p. R369*
P45	Female	61	ATM	c.4852C>T	p. R1618*

* means termination codon.

### 3.3 KRAS genetic diversity in PDCA

**Figure 4:**
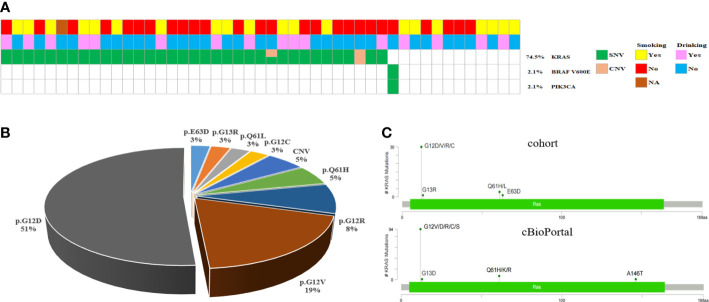
KRAS/BRAF-Pathway in PDAC. **(A)** Oncomap showing the distribution of KRAS, BRAF and PIK3CA mutation in the cohort. **(B)** KRAS mutations in the cohort exhibit a diverse spectrum concentrated at known oncogenic codons 12, 13, and 61. **(C)** Analysis of KRAS mutations in the PDAC cohort compared with all cancer cases in cBioPortal.

PDAC is represented by KRAS mutations, found in 74.5% (35/47) of the instances in our cohort, but 12 patients carrying wild-type KRAS. Whereas previous research has revealed that a small minority of PDAC patients harbored wild-type KRAS, the genomic drivers of these cases have remained obscure. Moreover, we noticed a PIK3CA mutation and a BRAF V600E mutation, both occurring in cases with wild-type KRAS (**Figure 4A**), consistent with another similar PDAC study (20). Another study claimed that different KRAS mutations had varying biological activities (21). KRAS activated mutations, mainly in codon 12 (G12D, G12V, and G12R), are regarded as the driver variation, and it is the first recurrently altered gene found in virtually all PDAC patients (20, 22). Consequently, the mutation locus distributions of KRAS were investigated further. The bulk of KRAS mutations were detected in codon 1 2, but they were also found in codons 13 and 61. Among these 25 KRAS mutation patients, the most frequent was G12D mutations, followed by G12V, G12R, G12C, Q61L, G13R and E63D (**Figure 4B**). Moreover, the mutation profiles of KRAS in the PDAC cohort were consistent with PDAC cases in cBioPortal (**Figure 4C**). These findings showed that genetic profiling of PDAC patients might identify a subset of individuals who potentially benefit from targeted treatment along the KRAS pathway.

### 3.4 A comparison of the genetic landscapes of several cohorts

**Figure 5:**
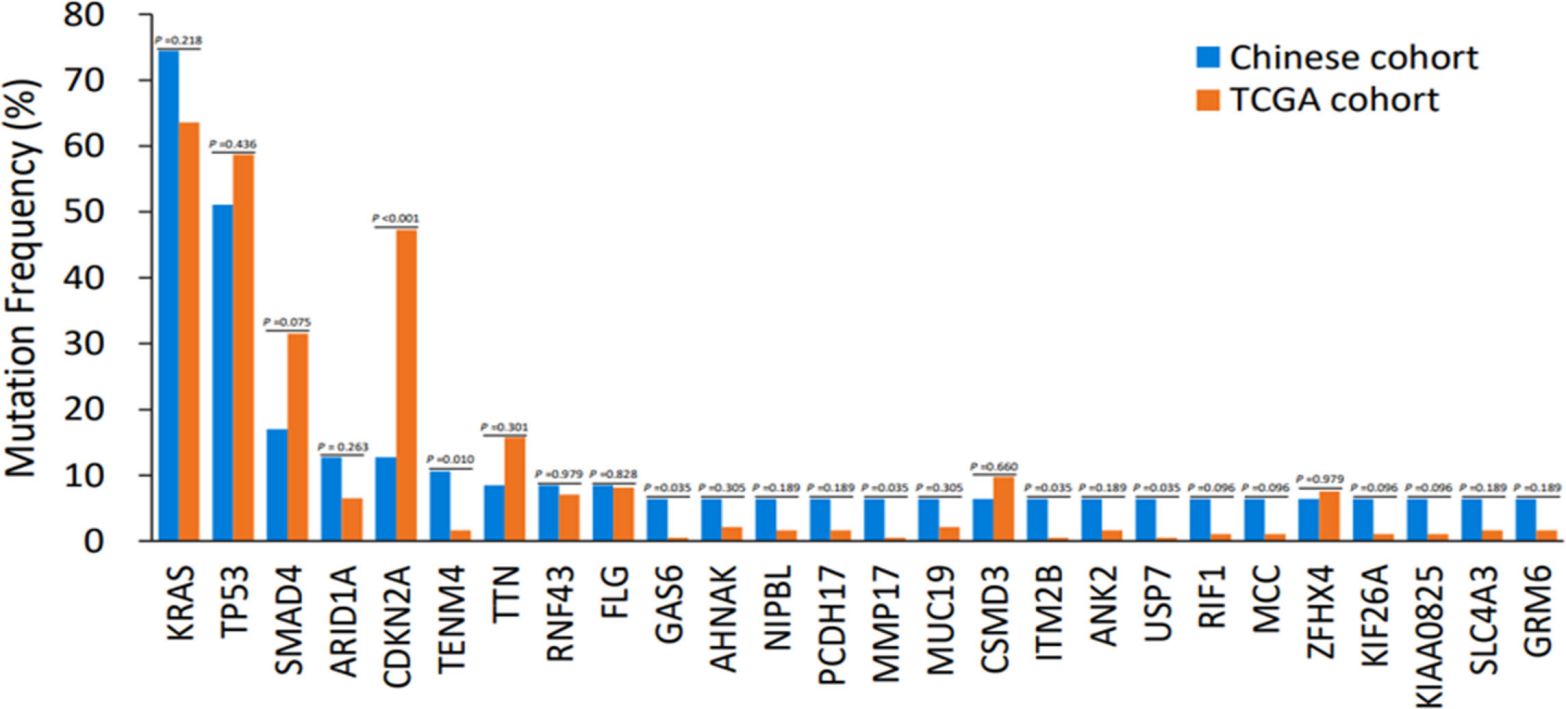
Discrepancies of mutation frequency between the Chinese cohort and TCGA dataset. p < 0.1, p < 0.05, p < 0.01. TCGA, The Cancer Genome Atlas.

To examine the unique genetic characteristics associated with Chinese PDAC, the WES genomic landscape was linked between the Chinese and TCGA database. Compared with TCGA PDAC cohorts, significantly fewer genetic changes were identified in SMAD4 (17.0% *vs*. 31.5%, *p* = 0.075) and CDKN2A (12.8% *vs*. 47.3%, *p* < 0.001), and significantly more mutations in TENM4(10.6% vs. 1.6%, *p* = 0.01), GAS6(6.4% *vs*. 0.5%, *p* = 0.035), MMP17(6.4% *vs*. 0.5%, *p* = 0.035), ITM2B (6.4% *vs*. 0.5%, *p* = 0.035) and USP7 (6.4% *vs*. 0.5%, *p* = 0.035) that were detected among Chinese PDAC cohorts. A potential statistical difference was noticed between the two cohorts. Although the KRAS mutation rate in Chinese cohorts was greater than in Western cohorts, no significant difference was found (74.5% *vs.* 63.6%, *P* =0.218) (**Figure 5**).

### 3.5 TMB and PD-L1 analysis in PDAC

**Figure 6:**
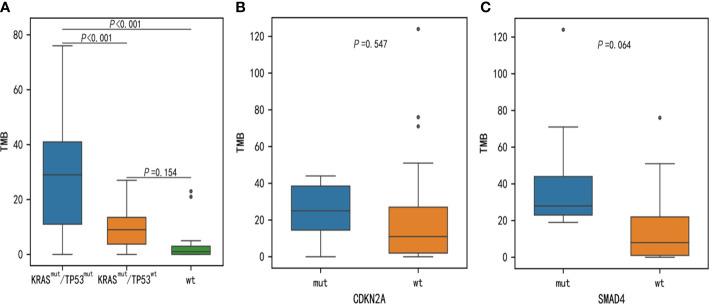
Discrepancies of TMB between KRAS, TP53, CDKN2A, and SMAD4 somatic alterations. TMB, tumor mutational burden.

**Figure 7:**
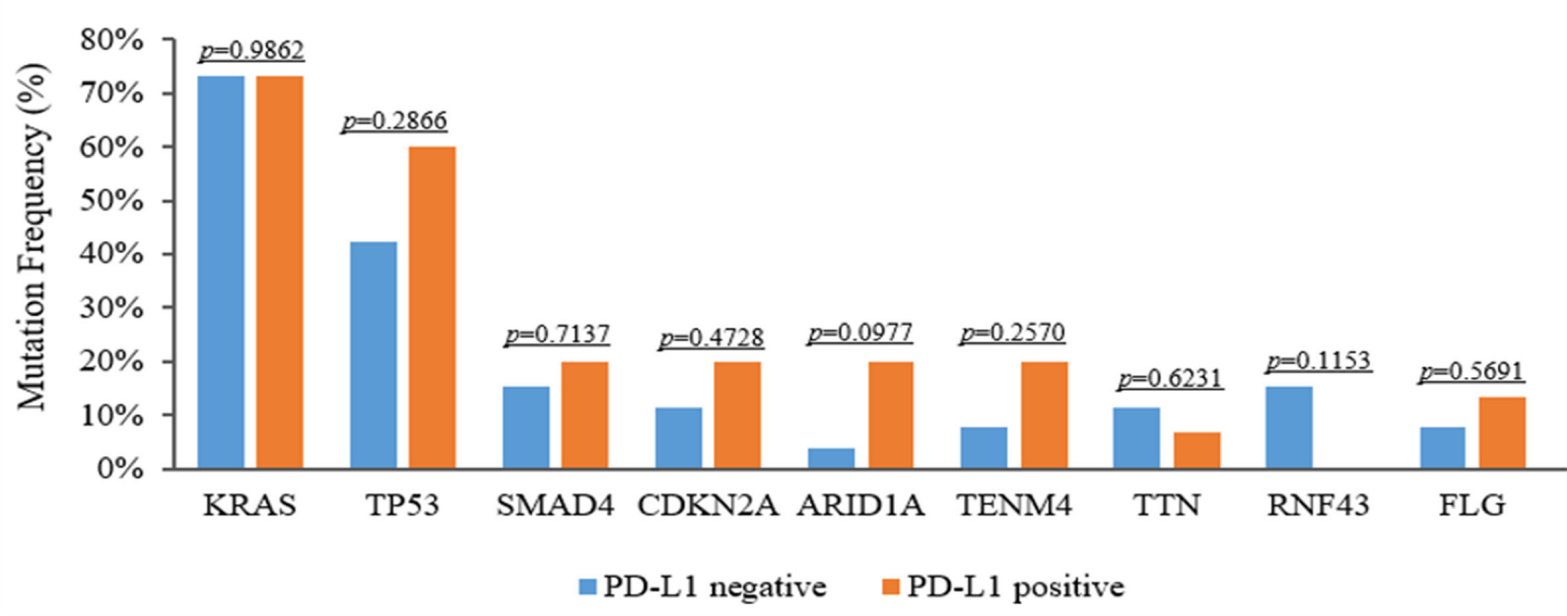
Discrepancies of mutation frequency between PD-L1-positive and PD-L1-negative patients.

It is well established that TMB is linked to the reaction of immunotherapy in certain forms of cancer. To evaluate genomic variations and their correlation with TMB, we analyzed these PDAC patients and determined that mutations KRAS, TP53, CDKN2A and SMAD4 were associated with high TMB. The TMB index was higher in patients with mutant-type KRAS/TP53 (*p* < 0.001), CDKN2A (*p* = 0.547), or SMAD4 (*p* = 0.064) compared to patients with wild-type KRAS/TP53, CDKN2A, or SMAD4 (**Figures 6A–C**). Meanwhile, we also assessed the relationship between PD-L1 expression and genomic variations. Although TP53 and TENM4 mutation rate in PD-L1-positive cohorts was higher than that in PD-L1-negative cohorts, no significant difference was found (*P* = 0.286 and *P* = 0.257). However, A possible statistical difference was detected in ARID1A between the two cohorts (*P* = 0.097, respectively) (**Figure 7**).

## 4 Discussion

Recently, various research efforts, such as the TCGA, have illustrated the landscape of genomic somatic mutations in PDAC, which might be useful for investigating oncogenesis and finding novel treatment methods. Although, racial disparities may also play a role in certain genetic somatic abnormalities in cancers. It is well established that foreign researchers unmasked most PDAC-related somatic mutations. The sufferers were mostly Caucasian and black. The similarities and differences in the somatic mutation landscapes in PDAC between Chinese patients and individuals from other regions and countries must still be investigated in a bigger sample population. Moreover, several studies have been evaluated using panel-based NGS involved in most of the vital genes in Chinese PDAC (10, 23). Some possibly beneficial genes might have been ignored. Combined and comprehensive whole-genome analyses need to be undertaken. Moreover, up until now, only 6 Chinese patients with PDAC have been previously examined by means of WES (13). In this investigation, WES was done on paired fresh-frozen tissues acquired from 47 PDAC patients. The current work highlights germline and somatic mutations found throughout the PDAC exome. Three germline deleterious variations in cancer susceptibility genes, known as pancreatic cancer susceptibility genes (ATM, WRN, and PALB2), were identified in our study population. Moreover, each of the three genes was linked to DNA damage repair (DDR) mutations. In accordance with recent investigations, a genetic association analysis involving 1,009 Chinese patients with PDAC detected 6.2% pathogenic sequence variations (24). Interestingly, no BRCA2 variants were identified in our cohort; perhaps the sample sizes were too small. Chinese PDAC patients with germline and somatic DDR mutations may be able to predict the efficacy of Olaparib and platinum-based chemotherapy, according to a retrospective study (12). Our findings suggested that patients with detected germline variants should undertake familial surveillance and screening. In addition, our research uncovered a potential link between DDR mutations and therapeutic effects, which may inspire additional biomarker studies focusing on impaired DNA pathways or immunotherapies.

The five most frequently mutated genes in 47 PDAC patients were KRAS (74.5%), TP53 (51.1%), SMAD4 (17%), ARID1A (12.8%), and CDKN2A (12.8%). Moreover, compared to data from the United States (25), the five most frequently mutated genes were KRAS (92%), TP53 (50%), SMAD4 (19%), FLG (10%), and ATXN1 (7%). Some genes (ARID1A, CDKN2A) in our cohort exhibited a higher mutation frequency. While TENM4, TTN, and GAS6 mutations were nearly undetectable in the American PDAC cohort, TENM4 mutations were nearly undetectable. ATXN1 mutations were also undetectable in our study. TENM4, TTN, FLG, and GAS6 were among the high-frequency variants in our study that, to the best of our knowledge, had not been previously described using panel-based NGS. Teneurin 4 (TENM4) is a transmembrane protein that is encoded by the ODZ4 gene and is engaged in neurite outgrowth, nervous system improvement, and neuronal differentiation. Recently, rearrangements and mutations in TENM4 have been found in various cancers. TENM4 expression is dysregulated in a variety of tumor types. However, the research on TENM4’s involvement as an oncogene or oncosuppressor is minimal and inconsistent (26–29). The latest information (GEPIA; http://gepia.cancer-pku.cn, accessed on 28 January 2021) showed a substantial elevation in TENM4 mRNA expression in human PDAC compared to healthy tissues. These data suggested that TENM4 might be an intriguing and appropriate target, not only for cancer of the pancreas, but for various types of tumors. More research is needed better to comprehend the role of TENM4 in PDAC development.

The TITIN (TTN) gene has not been extensively explored as a tumor-related gene in the literature, although it ranks eighth on our list. The TTN gene, which encodes titin protein, a critical element in the construction and function of vertebrates’ striated muscles (30), is often altered in main pancreatic cancer (31, 32). Moreover, previous studies demonstrated that TP53, SMAD4, KRAS, and TTN were the top four most frequently mutated genes in the TCGA PDAC cohort. However, there was no substantial difference in prognosis between non-mutated and mutated TTN tumors (33). In our data, four patients had five different TTN mutations (p.H33217Q, p.G26516S, p.G23637V, p.T32477P, p. L34705Ffs*42, p.R2320C). Moreover, three patients had pancreatic cancer with liver or abdominal metastasis.

FLG, also known as filaggrin, encodes a mesenchymal grin, a protein that collects keratin mesenchymal structures in the human epidermis. FLG is a tumor inhibitor gene drastically decreased in malignant tumors and is implicated in abnormal glucose absorption and mitochondrial alterations. FLG mutations can cause aberrant immunological responses, resulting in a variety of inflammatory illnesses. In a prior study, FLG was found to have a higher mutation frequency in prostate cancer and to be associated with an increase in TMB, up-regulation of aberrant immune responses in tumors, and cancer growth than the wild type (34). The recently published data showed that the FLG-mutant group (FLG-MT) had a greater mutation load and immunogenicity, suggesting that the gene mutation may be a preventative measure in gastric tumor (35). Other early results suggested that certain atopy-related variations, including the FLG gene may be linked to an increased risk of pancreatic cancer (36). In the present study, we identified 5 mutations (p.S535L, p.S2668A, p.S2512N, p.M58V, and p.Q3246L) in this gene, p.R579Q, in 4 PDCA patients.

Growth arrest-specific gene 6 (Gas6) is a multipurpose factor that affects various activities in both normal and pathological physiology (37). Gas6 and its major receptor Axl are abundantly expressed in various cancers, such as ovarian, breast, glioblastoma, gastric, lung, and pancreatic tumor, and their presence is associated with a bad prognosis (38). The Gas6-Axl pathway is active in 70percent of individuals with pancreatic cancer (39) and is linked to a poor prognosis and a rise in the number of distant metastases (40). Notably, GAS6 acts on both tumor cells and NK cells simultaneously, facilitating metastatic activities in pancreas cancer (41). Consequently, GAS6 has been proposed as a therapeutic target for pancreatic cancer. Blocking Gas6-Axl signaling, In PDCA sufferers, numerous Axl inhibitors and warfarin (a vitamin K antagonist that suppresses Gas6 signaling) are presently being explored (42, 43). In this investigation, we found just one mutation (p.L18Q) in this gene in three PDCA patients.

The key molecular event in PDAC individuals is KRAS mutation, which results in persistent stimulation of the KRAS protein, which acts as a genetic switch to stimulate numerous cellular signaling pathways and transcription factors, promoting propagation, infiltration, movement, and survivability (44). In our investigation, KRAS mutation was substantially associated with three tumor inhibitor genes, SMAD4, TP53, and CDKN2A. The aggregation of mutations in these four primary driver genes not only represents the distinctive molecular features of PDAC, but, is also linked to a bad prognosis (9). Moreover, several KRAS mutational subtypes were found in our cohort, with G12D accounting for 54percent of the total, similar to the mutation frequency described in the prior study. A prior study found that G12D mutant individuals with PDAC had a considerably shorter life expectancy than G12V, G12R, or wild-type individuals (45). Sotorasib (AMG510), a selective and irreversible small-molecule suppressor targeting the KRAS G12C mutation, has shown promising antitumor effects in non-small-cell lung cancer (NSCLC) (46), as well as other solid tumors, especially pancreatic cancers with the KRAS G12C mutation (47). In our cohort, only one case presented KRAS G12C mutation, and looked to benefit more from unique specific therapy approaches. Moreover, the bulk of PDAC modifications was KRAS G12D and G12V. In the previous investigation, the KRAS G12D mutant subtype is an independent predictive indicator for progressed pancreatic ductal cancer (45). Therefore, stopping the downstream pathway of KRAS appears to be a viable therapy option. We believe that new anti-KRAS agents might be conceived and created for the proper population of KRAS mutant patients in the future. In our analysis, the percentage of KRAS wild-type participants was 25.5%, significantly higher than reported in Western populations. Subjects with KRAS wild-type traditionally had a favorable prognosis (44).

The clinical significance and predictive value of PD-L1 in pancreatic cancer are still a matter of debate. PD-L1 served as a negative indicator for PDAC patients’ OS in a meta-analysis, and PD-L1 overexpression was associated with neural invasion and inadequate differentiation (48). In our research, 15 patients exhibited positive expression (TPS 1%), including two patients with extremely positive expressions. Moreover, these patients may have a dismal prognosis. ARID1A deficiency may be a novel predictive biomarker for treating immune checkpoint blockade (ICB). ARID1A deficiency would compromise the mismatch repair pathway and increase the number of tumor-infiltrating lymphocytes, tumor mutation burden, and expression of PD-L1 in a subset of malignancies, suggesting that ICB treatment would be more effective in these cases (49). We analyzed the relationship between PD-L1 expression and ARID1A variations in our cohort. PD-L1-positive cohorts had a higher ARID1A mutation rate than PD-L1-negative cohorts. This result suggested that ICB therapy approaches may be more beneficial for these patients. In conclusion, this work highlighted the features of WES clinical sequenced genomic profiles in real-world PDAC individuals from China. We anticipate that our results may aid in identifying potentially targetable and predicted biomarkers, as well as the investigation of clinical practice and new agent development for PDAC clients. However, there are also several limitations to this study. First, because of the limited size of our research group, the survival analyses and any clinical connections predicted might not be accurate. Greater PDCA cohort research is required to investigate possibly significant clinical relationships. Moreover, pharmacological profiling and prognostic information matching genomes are inadequate, and future clinical studies should be well-planned.

## Data availability statement

The raw data supporting the conclusions of this article will be made available by the authors, without undue reservation.

## Ethics statement

The human-participant research was evaluated and authorized by Medical Ethics Committee of the Second Affiliated Hospital of Army Medical University. The patients/participants provided their written informed consent to participate in this study.

## Author contributions

YH, YL and WH collected and organized the data. YT, CL and LL wrote the manuscript. XP, JW and JL prepared the figures. NY, LZ and XH critically revised the manuscript for intellectual content. All authors contributed to the article and approved the submitted version.
